# *Lactobacillus reuteri* HCM2 protects mice against Enterotoxigenic *Escherichia coli* through modulation of gut microbiota

**DOI:** 10.1038/s41598-018-35702-y

**Published:** 2018-11-30

**Authors:** Tianwei Wang, Kunling Teng, Gang Liu, Yayong Liu, Jie Zhang, Xin Zhang, Min Zhang, Yong Tao, Jin Zhong

**Affiliations:** 1 State Key Laboratory of Microbial Resources, Institute of Microbiology, Chinese Academy of Sciences, Beijing, 100101 China; 2 University of Chinese Academy of Sciences, Beijing, 100101 China; 3 Key Laboratory of Agro-Ecological Processes in Subtropical Region, Institute of Subtropical Agriculture, Chinese Academy of Sciences, Hunan, 410125 China; 4 LongDa Foodstuff Group Co., Ltd, Shandong Province, Laiyang, 265231 China

## Abstract

Enterotoxigenic *Escherichia coli* (ETEC) is a leading cause of infectious diarrhea in children and postweaning piglets. ETEC infection results in induced pro-inflammatory responses in intestinal epithelial cells and dysbiosis of intestinal microbiota. Here, a *Lactobacillus reuteri* strain, HCM2, isolated from a healthy piglet showed a high survival rate in the harsh gastrointestinal tract environment and inhibited the growth of ETEC and its adherence to intestinal epithelial cells. Pre-supplementation with *L*. *reuteri* HCM2 for 14 days reduced the ETEC load in the jejunum of ETEC-infected mice and prevented the disruption of intestinal morphology by ETEC. The colonic microbiota of mice with or without HCM2 pre-supplementation were analyzed, and this analysis revealed that HCM2 could prevent dysbiosis caused by ETEC infection by stabilizing the relative abundance of dominant bacteria. These results indicate that *L*. *reuteri* HCM2 has the potential to attenuate the effect of ETEC on the colonic microbiota in infected mice.

## Introduction

Enterotoxigenic *Escherichia coli* (ETEC) is a leading cause of infectious diarrhea in children from developing nations and is responsible for an estimated 3–5 million deaths annually in children under the age of five^1^. ETEC is also the main infectious agent of postweaning diarrhea in piglets and is responsible for 50% of piglet deaths worldwide annually^2^. The K88 (F4) fimbrial adhesin, heat-stable and heat-labile enterotoxins have been identified as important virulence factors leading to diarrheal diseases in piglets, and account for 93% of ETEC infections in piglets^3–5^. ETEC is known to adhere to the small intestinal epithelium and to secrete enterotoxins that alter the functions of enterocytes by increasing secretion, which leads to severe secretory diarrhea in pigs^3^. ETEC results in the loss of microvilli in the jejunum and promotes inflammation in a mouse model^6^. Ren *et al*. found that ETEC infection promotes the expression of pro-inflammatory cytokines through the activation of the NF-kB and MAPK pathways^6^. ETEC infection also induces the expression of intestinal IL-17 and causes dysbiosis of intestinal microbiota_,_ increasing the abundance of *Lactococcus lactis* subsp. *lactis* in infected mice^7^.

Antimicrobial growth promoters and therapeutic antibiotics are widely used in animal farming to prevent neonatal and postweaning diarrhea^8–10^. But they also potentially contribute to the accumulation of antimicrobial drug resistance genes in both pathogenic and non-pathogenic human bacteria, which will lead to serious public health problems^11^. As a result, the use of all antibiotics as growth promoters has been progressively banned from European agriculture since 2006 under Regulation 1831/2003/EEC^12^. Thus, there is an urgent need to develop “green antibiotics” that have a minimal ecological impact on the animal commensal and environmental microbiomes^11^. Among the non-antibiotic alternatives, probiotics seem to have the highest potential as they are efficient against pathogenic strains in animals^2^. A probiotic is defined as “a live microorganism that, when administered in adequate amounts, confers a health benefit on the host”^13^. Several reports have elucidated the role of probiotic bacteria in preventing and treating gastrointestinal diseases^5,14,15^. One example is *lactobacillus* whose potential benefits for human and animal health include the improvement of lactose intolerance, prevention of intestinal infection, modulation of the intestinal microbiota, reduction of serum cholesterol, stimulation of the immune system, anticarcinogenic action, and antioxidative effects^16–19^. For instance, *L*. *rhamnosus* plus *L*. *acidophilus* protected against *Shigella* infection by increasing antioxidant levels^16^. Gao *et al*. also found that *L*. *plantarum* could induce a high level of immune response, stimulate the growth of many intestinal *Lactobacillus* spp. and accelerate intestinal microbiota maturation^20^.

*L*. *reuteri* is a common species that inhabits the gastrointestinal tract of human, pig, hamster, mouse, rat, dog, sheep, cattle, and different birds^8,21,22^. Numerous studies have demonstrated that *L*. *reuteri* is resistant to gastric acid and bile, positively improves the performance of pigs, prevents diarrhea, alleviates stress, alters gastrointestinal microbiota, reduces the abundance of colibacillus and regulates the immune system^8,23–25^. *L*. *reuteri* can produce a variety of antimicrobial substances, such as lactic acid and bacteriocin reuterin^26^. *L*. *reuteri* also has the capacity to adhere to mucin and colonize on intestinal epithelial cells through cell surface proteins^27^. However, whether *L*. *reuteri* protects against ETEC by altering gut microbiota in mice is unknown.

In this study, an *L*. *reuteri* strain, HCM2, was isolated from the cecum content of a 6-week-old healthy piglet and showed good probiotic characteristics *in vitro*. Using an ETEC-infected specific-pathogen-free mouse model, we demonstrated that *L*. *reuteri* could protect against ETEC colonization and intestinal disruption caused by ETEC by positively affecting the intestinal microbiota.

## Results

### *L*. *reuteri* HCM2 inhibits the growth of ETEC and its ability to adhere to intestinal epithelial cells

**Figure 1:**
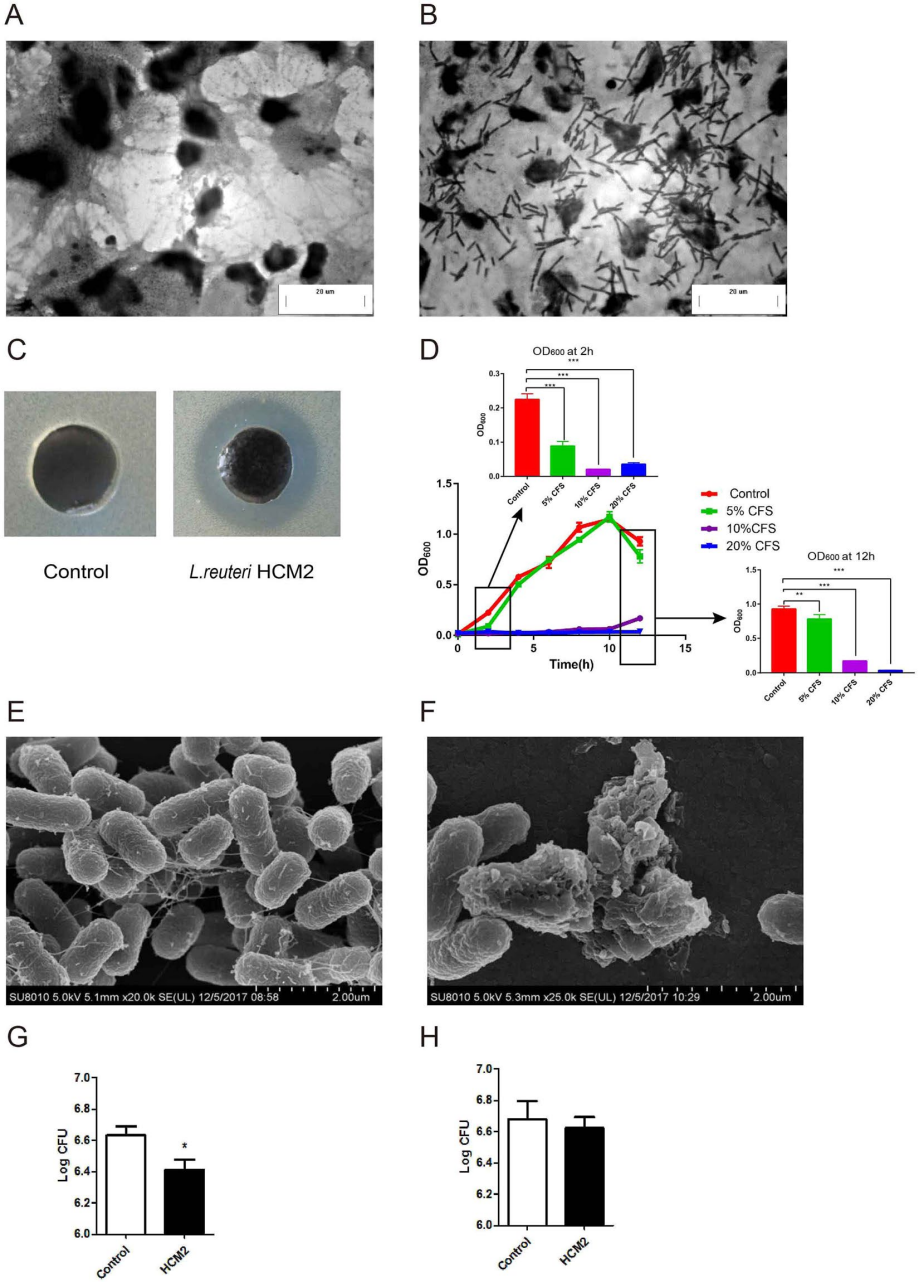
The probiotic properties of *L*. *reuteri* HCM2 (**A**) Caco-2 cells observed using light microscopy. (**B**) Adhesion of *L*. *reuteri* HCM2 to Caco-2 cells observed using light microscopy after Gram-staining. (**C**) An agar diffusion assay showing the antibacterial activity of *L*. *reuteri* HCM2 against ETEC. (**D**) The inhibition of ETEC growth by *L*. *reuteri* HCM2 was examined by adding different concentrations (5%, 10% and 20%) of *L*. *reuteri* HCM2 cell free supernatant (CFS) to ETEC inoculations and then measuring growth over 12 h. OD_600_ values of the inoculations at 2 h and 12 h are shown as bar graphs. The data are presented as mean ± SD with n = 5. (**E**) An SEM image of ETEC cells after 12 h of cultivation at 37 °C. (**F)**: An SEM image of ETEC cultivated in medium containing 20% *L*. *reuteri* HCM2-CFS for 12 h at 37 °C. The scale bar is 2 μm in (**E**,**F**). (**G**) Number of ETEC cells bound to Caco-2 cells after ETEC (10^8^ CFUs) and *L*. *reuteri* HCM2 (10^8^ CFUs) cells were incubated with Caco-2 cells at 37 °C for 90 min. H: Number of ETEC cells bound to Caco-2 cells after ETEC (10^8^ CFUs) cells were incubated with Caco-2 cells at 37 °C for 45 min, and then with *L*. *reuteri* HCM2 (10^8^ CFUs) and Caco-2 cells for another 45 min. These results include data from three independent experiments. *Significant difference at p < 0.05.

*L*. *reuteri* HCM2 showed a high rate of survival in artificial gastric juice (88.35%) and artificial small intestine fluid (73.93%) (Table 1). It could survive in bile salt concentrations of up to 0.3%, and the survival rates of *L*. *reuteri* HCM2 in 0.1% and 0.2% bile salt were 30.12% and 10.2%, respectively (data not shown). It also appeared to adhere to Caco-2 cells in chains when observed under a microscope (Fig. 1A,B); (16.1 ± 6.5) ×10^6^ colony forming units (CFUs) *L*. *reuter*i HCM2 cells were found to adhere to one square centimeter of Caco-2 cells. These results suggest that *L*. *reuteri* HCM2 is tolerant to the environment of the gastrointestinal tract and may adhere to the intestinal epithelial cells.

**Table 1:** Survival of *L*. *reuteri* HCM2 in artificial gastric juice (pH 2.5) and artificial small intestine fluid after incubation for 0 min and 2 h.

*L*. *reuteri* HCM2	Log CFU/ml (% survival)	
	0 min	2 h
Artificial gastric juice	7.54 ± 0.08 (100)	7.48 ± 0.10 (88.35)
Artificial small intestine fluid	8.10 ± 0.02 (100)	7.97 ± 0.05 (73.93)

We next tested the effect of *L*. *reuteri* HCM2 on ETEC growth. We found that the CFS (cell free supernatant) of *L*. *reuteri* HCM2 produced a clear zone with a diameter ≥14 mm in ETEC lawns (Fig. 1C), indicating that *L*. *reuteri* HCM2 may be capable of inhibiting the growth of ETEC. To test this, different concentrations (5%, 10% and 20%) of the CFS of *L*. *reuteri* HCM2 were added to the ETEC culture medium, and ETEC growth was monitored. ETEC was obviously inhibited by 10% and 20% *L*. *reuteri* HCM2 CFS (Fig. 1D). Observations under an SEM (scanning electron microscope) revealed that the CFS destroyed the rod-shaped structures of the ETEC cells (Fig. 1E,F). These results indicate that the CFS of *L*. *reuteri* HCM2 might inhibit ETEC growth by damaging the cell wall. ETEC cells had the ability to adhere to Caco-2 cells; however, the number of ETEC cells binding to Caco-2 cells was significantly reduced when ETEC were co-cultured with *L*. *reuteri* HCM2 (Fig. 1G). These results suggest that *L*. *reuteri* HCM2 could compete with ETEC cells and prevent them from adhering to Caco-2 cells. By contrast, no obvious difference in the number of ETEC cells bound to Caco-2 cells was observed when *L*. *reuteri* HCM2 cells were added to a pre-culture of ETEC and Caco-2 cells (Fig. 1H). Thus, it seems that *L*. *reuteri* HCM2 contributes little to replace ETEC that have already bound to Caco-2 cells. We hypothesized that *L*. *reuteri* HCM2 protects intestinal epithelial cells against ETEC.

### *L*. *reuteri* HCM2 reduces ETEC load in the jejunum and preserves intestinal morphology in mice

**Figure 2:**
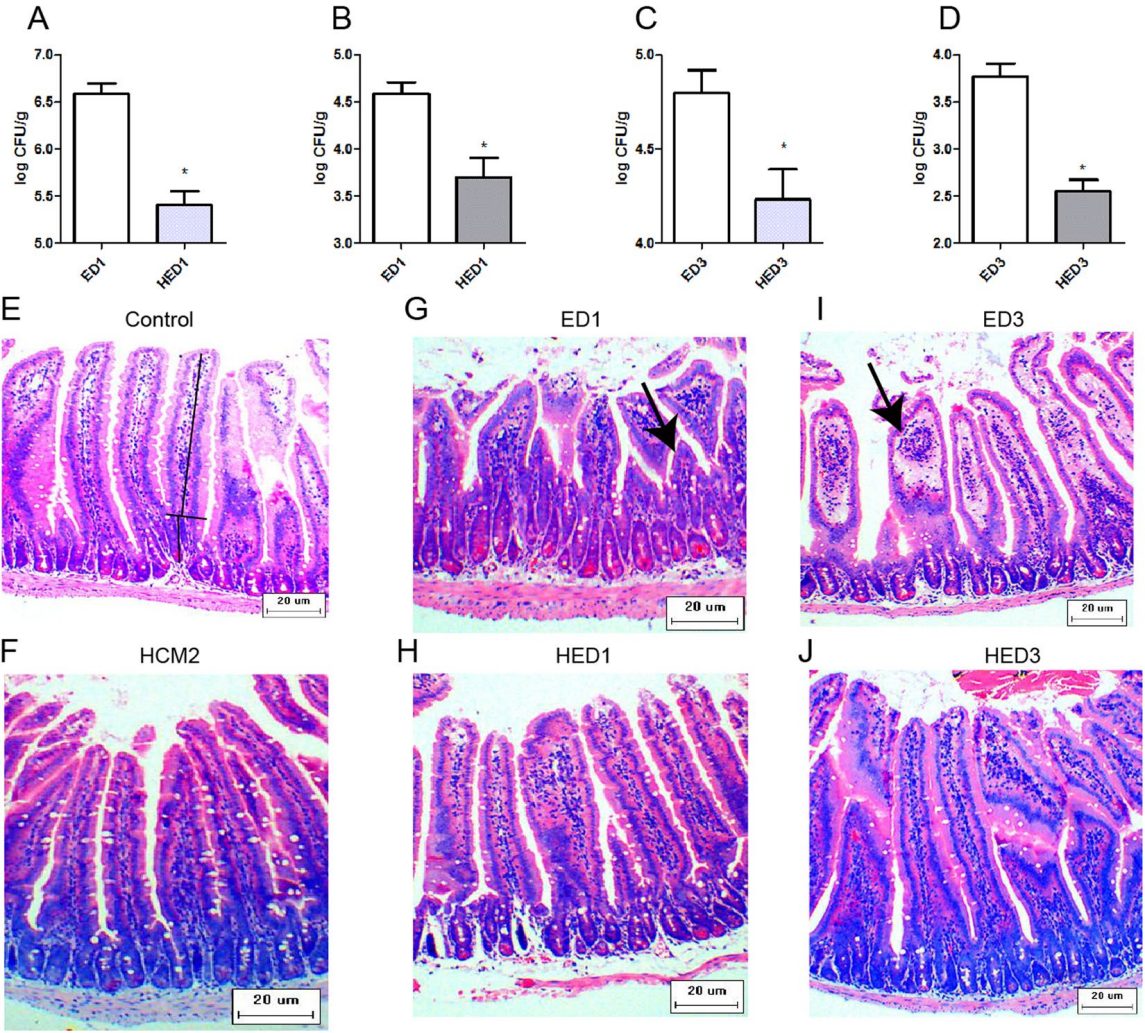
*L*. *reuteri* HCM2 preserves intestinal morphology in ETEC infected mice. (**A**) The load of ETEC in the jejunal tissues at D16; (**B**) The load of ETEC in the jejunal contents at D16; (**C**) The load of ETEC in the jejunal tissues at D18; (**D**) The load of ETEC in the jejunal contents at D18; E to J: Representative images of HE staining of the jejunum of weanling mice are shown (×100; n = 5). The villus length and crypt depth were measured as indicated in the image in (**E**). The black arrows in G and I indicate jejunal tissues that were damaged by ETEC. Control: mice received a basal diet. HCM2: mice received a basal diet and 10^9^ CFUs *L*. *reuteri* HCM2 daily for two consecutive weeks. ED1: mice received a basal diet, were challenged with 10^8^ CFUs ETEC by intragastric administration at day 15, then received a basal diet for 1 day. HED1: mice received a basal diet and 10^9^ CFUs *L*. *reuteri* HCM2 daily for two consecutive weeks, were challenged with 10^8^ CFUs ETEC by intragastric administration at day 15, and then received a basal diet for 1 day. ED3: mice received a basal diet, were challenged with 10^8^ CFUs ETEC by intragastric administration at day 15, then received a basal diet for 3 days. HED3: mice received a basal diet and 10^9^ CFUs *L*. *reuteri* HCM2 daily for two consecutive weeks, were challenged with 10^8^ CFUs ETEC by intragastric administration at day 15, and then received a basal diet for 3 days.

The number of ETEC CFUs in the jejunal tissues and jejunal contents were determined to estimate the ETEC load in mice. Before challenging mice with ETEC at day 15 (D15), no ETEC were detected in mice in the Control and HCM2 groups. The mice infected by ETEC all survived. At day 16 (D16), after 1 day of recovery from the ETEC challenge, the ETEC loads in the jejunal tissues (p < 0.05) and jejunal contents (p < 0.05) of mice pre-supplemented with *L*. *reuteri* HCM2 (HED1 group) were significantly lower than those in mice without *L*. *reuteri* HCM2 pre-treatment (ED1 group) (Fig. 2A,B). At day 18 (D18), after 3 days of recovery from the ETEC challenge, the ETEC loads in the jejunal contents and jejunal tissues of mice pre-supplemented with *L*. *reuteri* HCM2 for 14 days (HED3) were significantly lower (p < 0.05) than those in mice without *L*. *reuteri* HCM2 pre-treatment (ED3) (Fig. 2C,D). These results demonstrate that *L*. *reuteri* HCM2 can reduce ETEC loads in the mouse jejunum.

**Figure 3:**
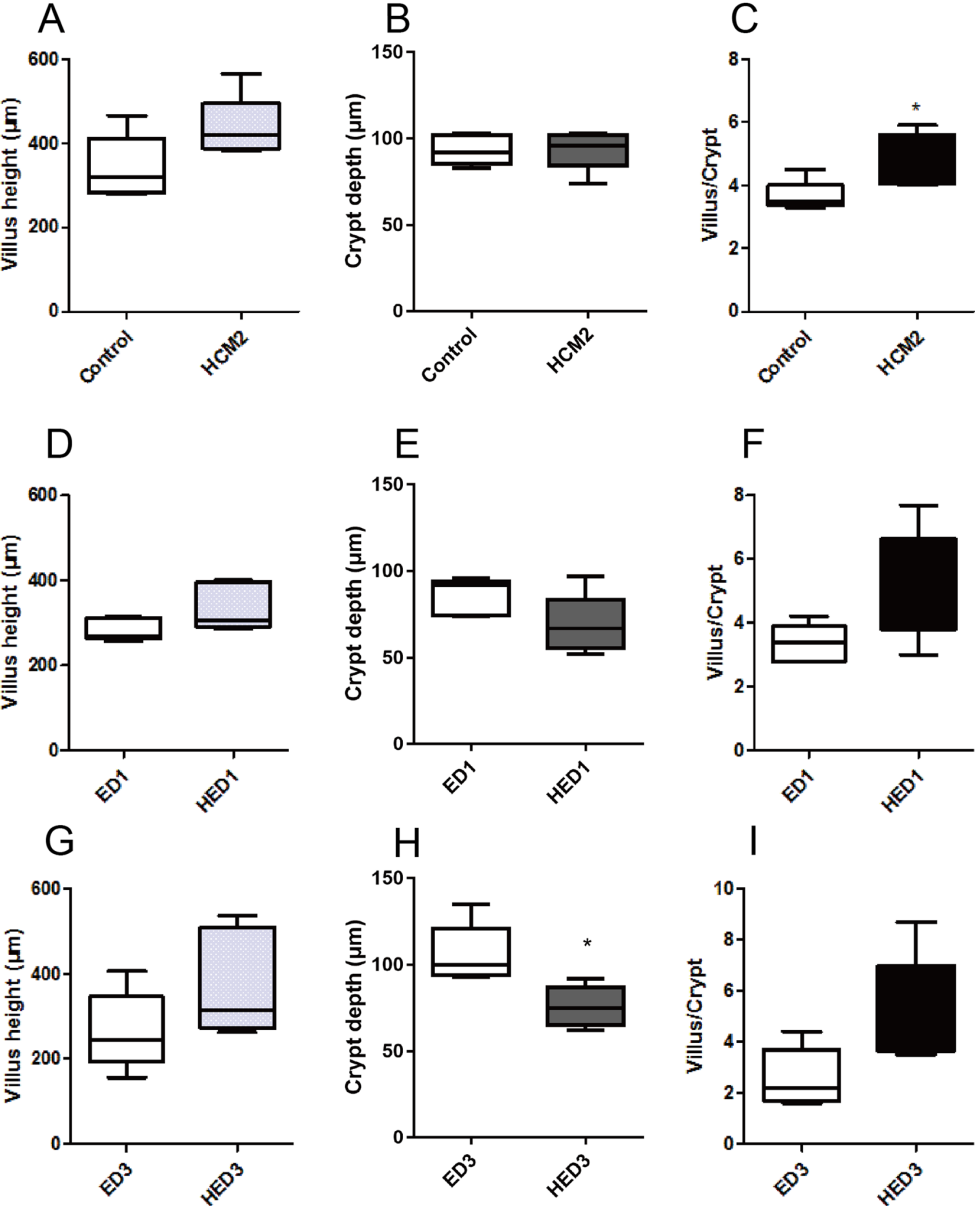
The villus length, crypt depth and the ratio of the villus to crypt in different groups of mice. (**A–C**) villus length (**A**), crypt depth (**B**), and the ratio of villus to crypt (**C**) at D15. (**D–F**) Villus length (**D**), crypt depth (**E**) and the villus to crypt ratio (**F**) at D16. (**G–I**) Villus length (**G**), crypt depth (**H**) and the villus to crypt ratio (**I**) at D18. The data are presented as mean ± SD with n = 5. *Indicates significance at p < 0.05. Control: mice received a basal diet. HCM2: mice received a basal diet and 10^9^ CFUs *L*. *reuteri* HCM2 daily for two consecutive weeks. ED1: mice received a basal diet, were challenged with 10^8^ CFUs ETEC by intragastric administration at day 15, then received basal diet for 1 day. HED1: mice received a basal diet and 10^9^ CFUs *L*. *reuteri* HCM2 daily for two consecutive weeks, were challenged with 10^8^ CFUs ETEC by intragastric administration at day 15, and then received a basal diet for 1 day. ED3: mice received a basal diet, were challenged with 10^8^ CFUs ETEC by intragastric administration at day 15, then received a basal diet for 3 days. HED3: mice received a basal diet and 10^9^ CFUs *L*. *reuteri* HCM2 daily for two consecutive weeks, were challenged with 10^8^ CFUs ETEC by intragastric administration at day 15, and then received a basal diet for 3 days.

**Figure 4:**
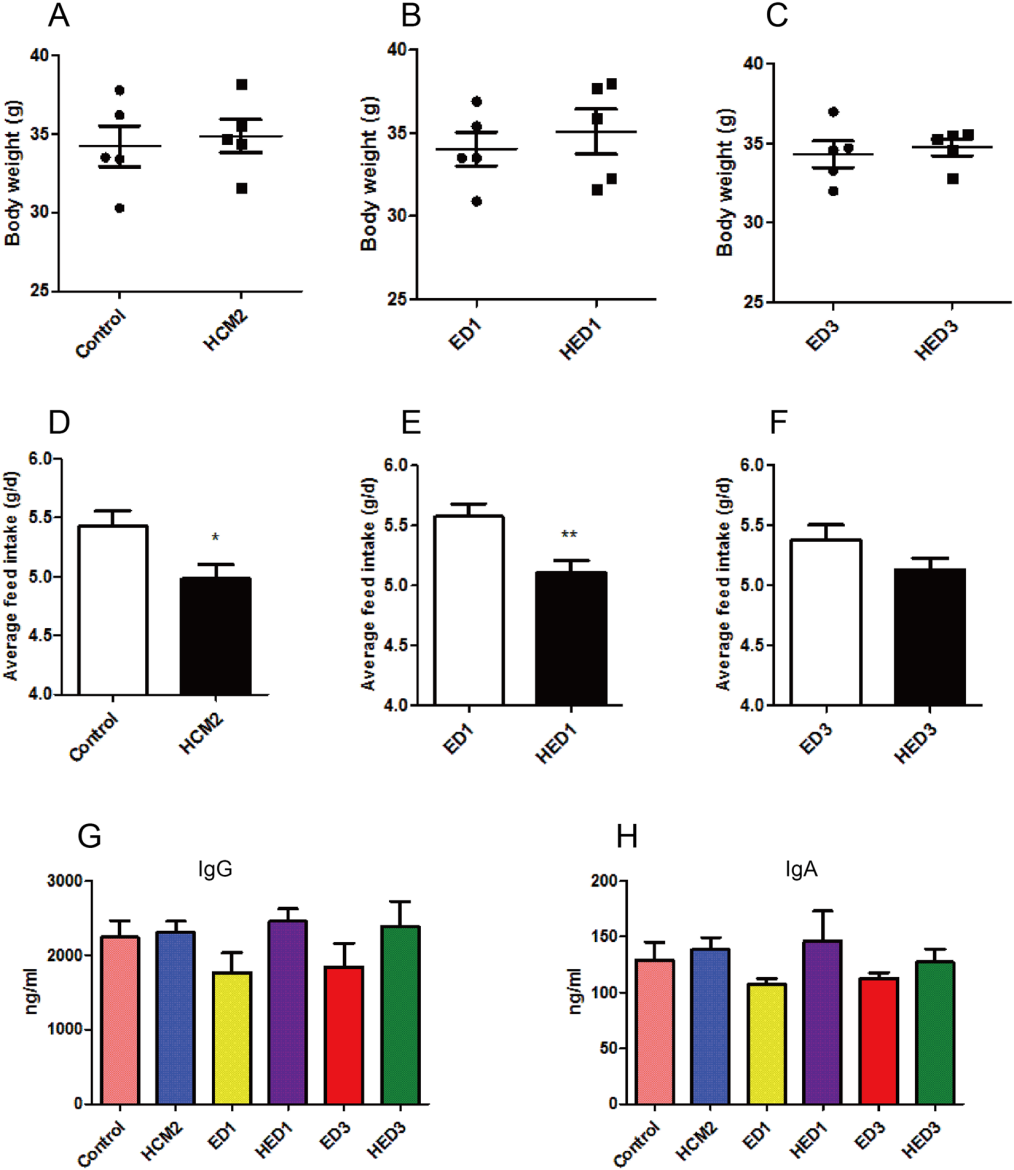
Body weight and feed intake for different groups of mice. (**A–C**) The body weight of mice in different groups. (**D–F**) The average feed intake for different groups. (**G**) The expression level of serum IgG in mice from different groups. (**H**) The expression level of serum IgA in mice from different groups. The data are presented as mean ± SD with n = 5. * indicates significance at p < 0.05. Control: mice received a basal diet. HCM2: mice received a basal diet and 10^9^ CFUs *L*. *reuteri* HCM2 daily for two consecutive weeks. ED1: mice received a basal diet, were challenged with 10^8^ CFUs ETEC by intragastric administration at day 15, then received a basal diet for 1 day. HED1: mice received a basal diet and 10^9^ CFUs *L*. *reuteri* HCM2 daily for two consecutive weeks, were challenged with 10^8^ CFUs ETEC by intragastric administration at day 15 and then received a basal diet for 1 day. ED3: mice received a basal diet, were challenged with 10^8^ CFUs ETEC by intragastric administration at day 15, then received a basal diet for 3 days. HED3: mice received a basal diet and 10^9^ CFUs *L*. *reuteri* HCM2 daily for two consecutive weeks, were challenged with 10^8^ CFUs ETEC by intragastric administration at day 15, and then received a basal diet for 3 days.

Morphological analyses revealed that ETEC infection led to inflammatory infiltration and loss of microvilli in the jejunum tissues of mice in the ED1 and ED3 groups (Fig. 2G,I), while inflammatory infiltration was not observed in mice in the HED1 and HED3 groups (Fig. 2H,J). These results indicate that *L*. *reuteri* HCM2 could protect the intestine from disruption by ETEC. At D15, mice pre-treated with *L*. *reuteri* HCM2 had a significantly higher villus/crypt ratio than mice in the control group (p < 0.05, Fig. 3C). At D18, three days after the ETEC challenge, the crypt depth was significantly lower in HED3 mice than in ED3 mice (p < 0.05, Fig. 3H). Although the feed intake at D15 (Fig. 4E) and D16 (Fig. 4F) was lower in mice receiving *L*. *reuteri* HCM2 supplementation, there was no obvious difference in body weight between groups (Fig. 4A–C). Levels of serum IgG and IgA, which reflect the system immune state^28^, were also determined and compared between the different groups. At D15, the HCM2 group had 2.9% and 7.67% higher serum IgG and IgA levels, respectively than the control group (Fig. 4G,H). At D16, the HED1 group had 38.93% and 35.63% higher levels of serum IgG and IgA, respectively, than the ED1 group (Fig. 4G,H). At D18, the HED3 group had 29.41% and 13.27% higher levels of serum IgG and IgA, respectively, than the ED3 group (Fig. 4G,H).

### Colonic microbiota diversity and composition analysis

The colonic microbiota of mice in the six groups were analyzed by sequencing the bacterial 16 S rRNA V3 + V4 region. High-throughput pyrosequencing of the samples (n = 5) produced a total of 2 757 344 raw reads. The effective reads were clustered into OTUs based on a similarity threshold of 97%. The 2 004 194 clean tags remaining after removing low-quality sequences were clustered into a total of 2247 OTUs, which were present in at least five samples. The rarefaction and rank abundance curves showed that the total richness of the microbial community was sampled completely (Fig. S1A,B). The Chao1, OS (Observed species), ACE, Shannon, Simpson, and PD (phylogenetic distance whole tree) indexes were calculated (Table S1). Based on these indexes there was not a big difference in microbial structure between mice receiving the *L*. *reuteri* HCM2 pre-treatment and those that did not.

The sequences across all samples were assigned to 27 phyla and 280 genera. *Firmicutes* and *Bacteroidetes* were the two predominant phyla in the mice gut microbiota, followed by *Proteobacteria*, *Actinobacteria*, and *Saccharibacteria* (Table 2). To investigate the gut bacteria in each group of mice, namely Control, HCM2, ED1, HED1, ED3 and HED3, the relative abundance of bacteria in each group was estimated based on the number of representative OTUs. The dominant sequences, comprising > 1% of the total bacteria composition, belonged to 22 genera: *Lactobacillus*, *Lachnospiraceae-_*NK4A136_group, C*andidatus Arthomitus*, *Ruminococcaceae_*UCG-014, unidentified_Lachnospiraceae, *Roseburia*, *Anaerotruncus*, *unidentified_Ruminococcaceae*, *Lachnospiraceae_*UCG-006, *Lachnoclostridium* and *Ruminiclostridium_*9, which belong to *Firmicutes*; *Alloprevotella*, *Alistipes*, *Bacteroides*, *Odoribacter*, *Parabacteroides*, *Rikenella* and *Rikenellaceae_*RC9_gut_group which belong to *Bacteroidete*; *Helicobacter* and *Desulfovibrio* which belong to *Proteobacteria*; *Bifidobacterium*, which belong to *Actinobacteria*; and *Candidatus Saccharimonas*, which belong to *Saccharibacteria*. We observed significant differences in the composition of microbiota between groups. Major changes in composition were mainly observed for *Firmicutes*, *Bacteroidete*, and *Proteobacteria*. Changes in the abundance of *Firmicutes* were mainly attributable to *Lactobacillus*. The relative abundances of all genera belonging to *Bacteroides* changed at D16, especially in the HED1 group. Changes in the abundance of *Proteobacteria* were mainly attributable to *Helicobacter* (Table 2).

**Table 2:** Relative abundance (%) of bacterial genera in the colonic microbiota of mice during ETEC treatment, determined by Illumina sequencing of 16S rRNA tags.

Genus	D15		D16		D18	
	Control	HCM2	ED1	HED1	ED3	HED3
**Firmicutes**						
*Lactobacillus*	75.08 ± 10.5^ab^	86.06 ± 3.75^a^	62.89 ± 10.1^bc^	44.63 ± 23.13^c^	52.76 ± 25.68^c^	86.27 ± 3.57^a^
*Lachnospiraceae_NK4A136_group*	0.75 ± 1.02	0.35 ± 0.29	1.13 ± 0.56	1.68 ± 2.26	2.4 ± 2.93	0.35 ± 0.21
*Candidatus_Arthromitus*	1.98 ± 0.69	2.19 ± 1.88	1.45 ± 0.69	1.85 ± 0.49	1.94 ± 2.77	1.4 ± 0.52
*Ruminococcaceae_UCG-014*	1.3 ± 0.6^bc^	0.64 ± 0.61^c^	1.02 ± 0.23^c^	2.68 ± 1.82^a^	1 ± 0.67^c^	1.18 ± 0.97^c^
*unidentified_Lachnospiraceae*	0.03 ± 0.01^b^	0.04 ± 0.04^b^	0.17 ± 0.05^b^	0.17 ± 0.26^b^	1.19 ± 1.66^a^	0.08 ± 0.05^b^
*Roseburia*	0.13 ± 0.12^ab^	0.07 ± 0.05^b^	0.19 ± 0.09^ab^	0.19 ± 0.27^ab^	0.81 ± 1.25^a^	0.09 ± 0.08^b^
*Anaerotruncus*	0.14 ± 0.06^b^	0.04 ± 0.01^b^	0.18 ± 0.07^ab^	0.3 ± 0.4^ab^	0.7 ± 0.95^a^	0.08 ± 0.06^ab^
*unidentified_Ruminococcaceae*	0.05 ± 0.03^b^	0.03 ± 0.01^b^	0.11 ± 0.06^ab^	0.03 ± 0.02^b^	0.57 ± 0.95^a^	0.02 ± 0.01^b^
*Lachnospiraceae_UCG-006*	0.06 ± 0.02	0.03 ± 0.01	0.11 ± 0.04	0.52 ± 0.92	0.11 ± 0.04	0.11 ± 0.09
*Lachnoclostridium*	0.16 ± 0.15	0.11 ± 0.06	0.14 ± 0.06	0.49 ± 0.86	0.27 ± 0.25	0.09 ± 0.07
*Ruminiclostridium_9*	0.15 ± 0.08^b^	0.06 ± 0.05^b^	0.2 ± 0.06^ab^	0.19 ± 0.13^ab^	0.47 ± 0.56^a^	0.07 ± 0.03^b^
*Coprococcus_1*	0.02 ± 0.01	0.01 ± 0.01	0.13 ± 0.11	0.1 ± 0.11	0.3 ± 0.55	0.01 ± 0.01
*Ruminiclostridium*	0.03 ± 0.01^b^	0.01 ± 0.01^b^	0.08 ± 0.02^ab^	0.03 ± 0.03^b^	0.32 ± 0.49^a^	0.01 ± 0.08^b^
*Oscillibacter*	0.03 ± 0.02^b^	0.01 ± 0.01^b^	0.05 ± 0.02^ab^	0.04 ± 0.05^ab^	0.24 ± 0.38^a^	0.002 ± 0.004^b^
*Clostridium_sensu_stricto_1*	0.22 ± 0.35	0.04 ± 0.02	0.05 ± 0.02	0.05 ± 0.02	0.04 ± 0.03	0.04 ± 0.02
*Staphylococcus*	0.04 ± 0.02^c^	0.03 ± 0.02^c^	0.09 ± 0.01^bc^	0.02 ± 0.01^c^	0.27 ± 0.32^a^	0.24 ± 0.01^ab^
*Erysipelatoclostridium*	0.08 ± 0.04	0.06 ± 0.08	0.56 ± 1.01	0.29 ± 0.31	0.19 ± 0.27	0.07 ± 0.08
*Allobaculum*	ND	0.02 ± 0.42	ND	ND	ND	0.01 ± 0.01
*Other (P: Firmicutes)*	3.44 ± 0.79^abc^	2.36 ± 0.81^c^	3.76 ± 1.15^ab^	2.94 ± 0.55^bc^	4.59 ± 1.34^a^	3.65 ± 0.95^ab^
***Bacteroidetes***						
*Alloprevotella*	0.21 ± 0.16	0.1 ± 0.1	1.79 ± 2.44	6.61 ± 12.04	0.73 ± 0.64	0.06 ± 0.04
*Alistipes*	1.33 ± 1.06^b^	0.23 ± 0.22^b^	1.77 ± 1.27^b^	5.79 ± 5.72^a^	1.44 ± 1.4^b^	0.24 ± 0.12^b^
*Bacteroides*	0.05 ± 0.03^b^	0.05 ± 0.04^b^	1.67 ± 0.98^ab^	2.89 ± 3.49^a^	0.28 ± 0.19^b^	0.07 ± 0.05^b^
*Odoribacter*	0.64 ± 0.45^ab^	0.26 ± 0.36^b^	0.98 ± 0.62^ab^	2.22 ± 2.55^a^	1.49 ± 2.01^ab^	0.11 ± 0.05^b^
*Parabacteroides*	0.09 ± 0.09^b^	0.02 ± 0.03^b^	0.41 ± 0.32^ab^	1.11 ± 1.36^a^	0.09 ± 0.09^b^	0.02 ± 0.01^b^
*Rikenella*	0.13 ± 0.12^b^	0.02 ± 0.02^b^	0.37 ± 0.29^ab^	0.66 ± 0.86^a^	0.14 ± 0.12^b^	0.03 ± 0.01^b^
*Rikenellaceae_RC9_gut_group*	0.34 ± 0.11^bc^	0.24 ± 0.09^c^	1.02 ± 0.22^a^	0.97 ± 0.28^a^	0.55 ± 0.19^b^	0.38 ± 0.01^bc^
*Other (P: Bacteroidetes)*	0.17 ± 0.04^c^	0.27 ± 0.29^bc^	0.86 ± 0.31^a^	0.61 ± 0.09^ab^	0.53 ± 0.5^abc^	0.2 ± 0.07^c^
***Proteobacteria***						
*Helicobacter*	4.34 ± 4.31^b^	0.14 ± 0.09^b^	6.93 ± 4.27^b^	1.8 ± 1.79^b^	13.59 ± 15.7^a^	0.42 ± 0.14^b^
*Desulfovibrio*	1.63 ± 1.97^a^	0.24 ± 0.17^b^	0.31 ± 0.14^b^	0.41 ± 0.36^b^	0.9 ± 0.97^ab^	0.37 ± 0.23^b^
*Acinetobacter*	0.02 ± 0.01	0.02 ± 0.01	0.03 ± 0.01	0.01 ± 0.01	0.23 ± 0.46	0.21 ± 0.01
*[F:Enterobacteriaceae]*	0.003 ± 0.00^b^1	0.001 ± 0.00^c^1	0.039 ± 0.05^a^a	0.008 ± 0.009^ab^b	0.042 ± 0.075^a^a	0.003 ± 0.003^b^c
*Other (P: Proteobacteria)*	0.15 ± 0.04	0.12 ± 0.04	0.19 ± 0.1	0.13 ± 0.08	0.16 ± 0.05	0.12 ± 0.04
***Actinobacteria***						
*Bifidobacterium*	0.06 ± 0.02	0.87 ± 1.76	ND	ND	0.04 ± 0.04	0.05 ± 0.02
**Saccharibacteria**						
Candidatus_Saccharimonas	1.43 ± 0.38^ab^	0.14 ± 0.06^b^	0.98 ± 0.61^ab^	2.08 ± 3.29^a^	1.48 ± 0.88^ab^	0.4 ± 0.5^ab^
**Tenericutes**						
[C:Mollicutes]	0.17 ± 0.16	0.07 ± 0.08	0.12 ± 0.09	0.1 ± 0.07	0.05 ± 0.03	0.03 ± 0.02
**Spirochaetes**						
[F:Spirochaetaceae]	0.01 ± 0.01	0.02 ± 0.01	0.01 ± 0.01	0.02 ± 0.01	0.01 ± 0.02	0.01 ± 0.01
**Others**	5.5 ± 2.65^b^	4.84 ± 2.63^b^	10.22 ± 4.82^b^	18.38 ± 8.68^a^	10.09 ± 7.11^b^	3.48 ± 1.02^b^
**Total**	99.98	100.00	99.96	99.99	99.96	99.99

Data are presented as means ± standard deviation of values at D15 (Control and HCM2), D16 (ED1 and HED1), and D18 (ED3 and HED3). Data in the same row that do not share a common superscript are significantly different (p < 0.05).

ND, not detected.

“Others” means the assignment is ambiguous.

### *L*. *reuteri* HCM2 stabilized the gut microbiota of mice challenged with ETEC

**Figure 5:**
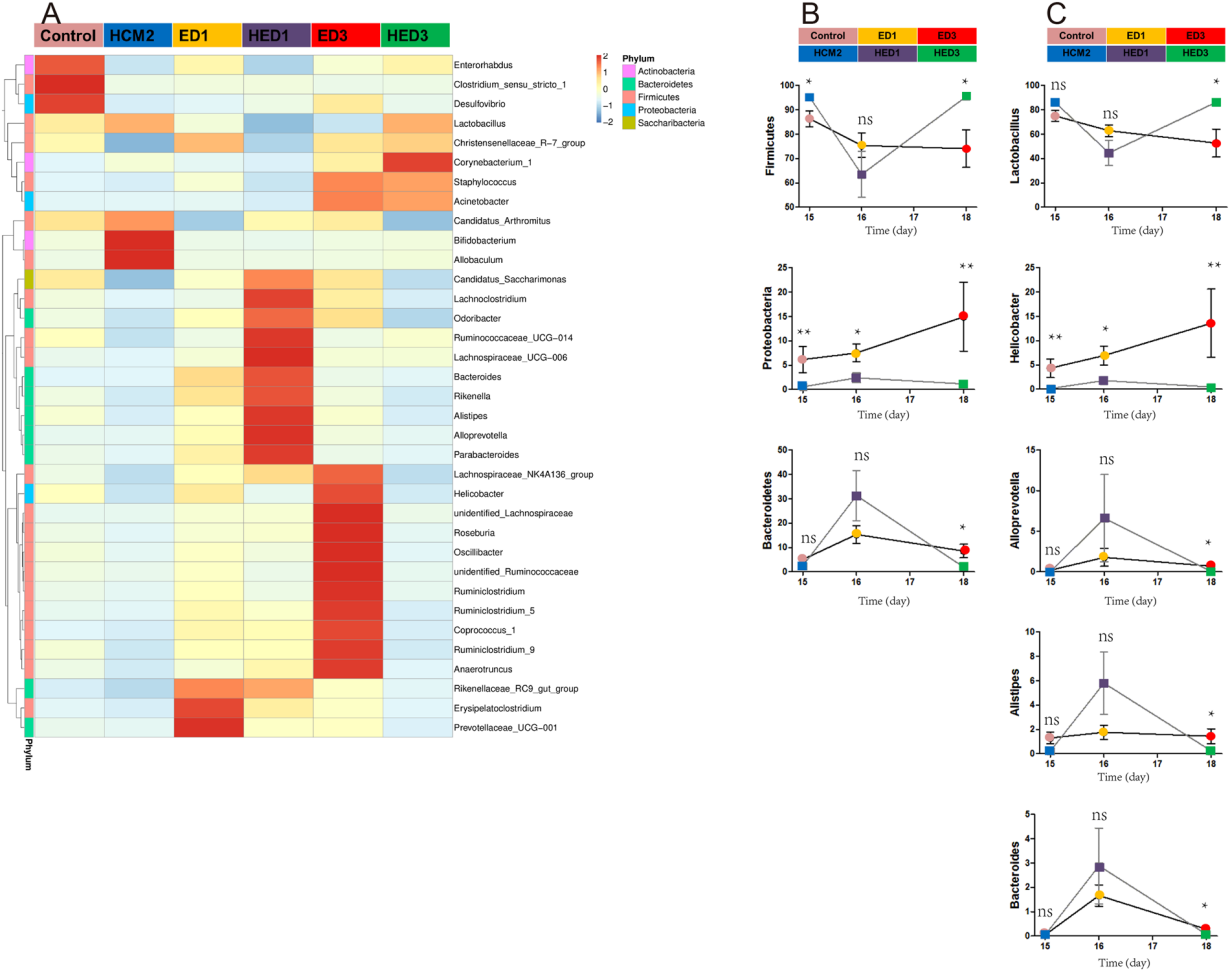
A heatmap of bacterial genera and dynamic changes in the dominant genera in each group. (**A**) A heatmap showing the abundances of bacterial genera in different groups. Relative abundance is scaled by the relative abundance within a genus. The color indicates the relative abundance as shown in the legend provided in the top right. (**B**) The relative abundances (mean ± SD) of the phyla *Firmicutes*, *Bacteroidetes* and *Proteobacteria* were plotted against time for each treatment. (**C**) The relative abundances (mean ± SD) of the genera *Lactobacillus*, *Allprevotella*, *Alistipes*, *Bacteroides* and *Helicobacter* were plotted against time for each treatment. * indicates significance at p < 0.05 for a single time point. Control: mice received a basal diet. HCM2: mice received a basal diet and 10^9^ CFUs *L*. *reuteri* HCM2 daily for two consecutive weeks. ED1: mice received a basal diet, were challenged with 10^8^ CFUs ETEC by intragastric administration at day 15, then received a basal diet for 1 day. HED1: mice received a basal diet and 10^9^ CFUs *L*. *reuteri* HCM2 daily for two consecutive weeks, were challenged with 10^8^ CFUs ETEC by intragastric administration at day 15 and then received a basal diet for 1 day. ED3: mice received a basal diet, were challenged with 10^8^ CFUs ETEC by intragastric administration at day 15, then received a basal diet for 3 days. HED3: mice received a basal diet and 10^9^ CFUs *L*. *reuteri* HCM2 daily for two consecutive weeks, were challenged with 10^8^ CFUs ETEC by intragastric administration at day 15, and then received a basal diet for 3 days.

The colonic microbiome was characterized by analyzing the relative abundance of bacterial taxa (Fig. 5A). The dynamic changes in several dominant phyla in each group of mice are shown in Fig. 5B. After ETEC challenge, the proportion of *Firmicutes* in mice without HCM2 supplementation (Control group) decreased from 86.4% to 75.4% after 1 day of recovery (ED1 group) and then to 74.2% after 3 days of recovery (ED3 group). This result indicates that ETEC challenge could reduce the proportion of *Firmicutes* in the mouse colon. The proportion of *Firmicutes* in mice supplemented with HCM2 decreased from 95.2% to 63.4% 1 day after ETEC challenge (HED1 group), but increased to the original level of 95.4% 3 days after ETEC challenge (HED3 group). This result demonstrates that although ETEC infection leads to a decreased proportion of *Firmicutes* in the short term, pre-treatment with *L*. *reuteri* HCM2 could help *Firmicutes* recover to the pre-infection level in the gut. Supplementation with *L*. *reuteri* HCM2 in advance also contributed to the recovery of *Bacteroidetes* to the pre-infection level in the gut 3 days after ETEC challenge. ETEC challenge led to an increase in the proportion of *Proteobacteria* from 6.2% in the control group to 7.4% in the ED1 group, and this proportion increased to 15% in the ED3 group. The proportion of *Proteobacteria* was different in the *L*. *reuteri* HCM2 supplementation groups. It increased from 0.4% to 2.4% in the HED1 group, and then decreased to 1.2% in the HED3 group (Fig. 5B). As shown in Table 2, after ETEC challenge, the proportion of *Enterobacteriaceae* in mice without HCM2 supplementation increased from 0.003% in the Control group to 0.039% in the ED1 group and then to 0.042% in the ED3 group. This result demonstrates that ETEC challenge could increase the proportion of *Enterobacteriaceae* in the mouse colon. The proportion of *Enterobacteriaceae* in mice supplemented with HCM2 increased from 0.001% in the Control to 0.008% 1 day after ETEC challenge (HED1 group), but decreased to pre-infection proportion of 0.003% 3 days after ETEC challenge (HED3 group). This result demonstrates that although ETEC infection leads to an increased proportion of *Enterobacteriaceae* in the short term, pre-treatment with *L*. *reuteri* HCM2 could help *Enterobacteriaceae* recover to the pre-infection level in the gut. Taken together, these results indicate that pretreatment of mice with *L*. *reuteri* HCM2 for 14 days before ETEC challenge could alleviate the disruption of the bacterial community caused by ETEC infection and help the gut microbiota to recover to pre-infection levels.

The dynamic changes in the dominant genera in each group of mice were also analyzed. *Lactobacillus* was the predominant genera in the mouse gut microbiota and showed a similar dynamic trend as *Firmicutes* after ETEC challenge (Fig. 5C). This result suggests that *Lactobacillus* might be the main genus contributing to the changes in *Firmicutes* after ETEC challenge. The proportion of bacteria in the *Helicobacter* genus also increased with increasing time after infection in groups without *L*. *reuteri* HCM2 supplementation and fluctuated in groups with *L*. *reuteri* HCM2 supplementation, a trend similar to that observed for *Proteobacteria*. This result suggests that *Helicobacter* might be the main genus contributing to the changes in *Proteobacteria* (Fig. 5C). The relative abundance of *Alloprevotella*, which belongs to the phylum *Bacteroidetes*, increased from 0.1% in the HCM2 group to 6.6% in the HED1 group, and then decreased to 0.1% in the HED3 group (Fig. 5C). Two other dominant genera, *Alistipes* and *Bacteroides*, which also belong to the phylum *Bacteroidetes*, showed similar dynamic trends as the phylum *Bacteroidetes* (Fig. 5C).

We next compared the colonic microbiota of mice in groups ED3 and HED3 with those of mice in the Control group using the linear discriminant analysis (LDA) effect size (LEfSe) method. A cladogram representing the structure of the colonic microbiota and the predominant bacteria taxa is shown in Fig. S2. Compared with the Control group, mice in the HED3 group had a significantly higher abundance of *Lactobacillus*, while mice in the ED3 group had an increased abundance of *Bacteroidia*, *Lachnospiraceae*, *Campylobacterales* and *Helicobacter* (Fig. S2A,B). These results demonstrate that *L*. *reuteri* HCM2 could protect mice from ETEC challenge by modulating the gut microbiota, restoring the pre-infection structure and promoting high levels of probiotic bacteria.

## Discussion

*L*. *reuteri* is a common species that inhabits the gastrointestinal tract of humans and many animals. Here we demonstrated that an *L*. *reuteri* HCM2 strain isolated from a healthy piglet could protect against ETEC infection in a mouse model by altering gut microbiota. The growth of ETEC was obviously inhibited by *L*. *reuteri* HCM2, and this might be caused by damage to the ETEC cell wall. As we did not observe any antimicrobial peptide production by *L*. *reuteri* HCM2, it seems that lactic acid might function as the antimicrobial agent inhibiting ETEC growth (data not shown). Competition for adhesion sites is one of the mechanisms by which probiotics fight against pathogen infection^29,30^. We found that *L*. *reuteri* HCM2 could survive in the harsh gastrointestinal tract environment and had the capacity to adhere to Caco-2 cells. Furthermore, a competition assay revealed that *L*. *reuteri* HCM2 was able to reduce the number of ETEC cells. These results demonstrate that *L*. *reuteri* HCM2 potentially protects against ETEC. We further observed that the ETEC load in both the jejunum tissues and contents of the mouse model was significantly decreased after *L*. *reuteri* HCM2 pre-supplementation. A similar phenomenon was also observed by Yang *et al*., who found that *L*. *reuteri* TMW1.656 could decrease the abundance of *E*. *coli* and K88 fimbriae in ETEC-challenged weanling pigs^31^.

Probiotics are important because they protect the host gastrointestinal micro-environment from invading pathogens^2^. In this study, ETEC infection led to inflammatory infiltration and loss of microvilli in jejunum tissues in mice not receiving *L*. *reuteri* HCM2 pre-supplementation (ED1 and ED3 groups). This is consistent with the finding of a previous study that ETEC infection promotes the expression of pro-inflammatory cytokines by activating the NF-kB and MAPK pathways, leading to the loss of microvilli in the jejunum^6^. However, inflammatory infiltration was not found in mice of the HED1 and HED3 groups, which were pre-supplemented with *L*. *reuteri* HCM2. The expression levels of IgA and IgG did not obviously increase in mice pre-supplemented with *L*. *reuteri* HCM2 compared with control mice. Gao *et al*. also reported that supplementation with *L*. *plantarum* did not obviously increase the expression levels of IgG in broiler chickens^20^, which supports our finding.

Several recent studies have noted that supplementation with *L*. *reuteri* improves the growth and feed efficiency of neonatal and growing pigs^8^. For example, Liu *et al*. reported that *L*. *reuteri* I5007 supplementation increased the average daily weight gain in formula-fed piglets^18^. Wang *et al*. found that pigs supplemented with *L*. *fermentum* I5007 had higher weight gain and feed intake than pigs without *L*. *fermentum* I5007 treatment^32^. In our study, mice supplemented with *L*. *reuteri* HCM2 took in less food, however, no significant difference in body weight was detected between control mice and mice supplemented with *L*. *reuteri* HCM2. *L*. *reuteri* HCM2 slightly decreased the feed conversion in HCM2 and HED1 mice (Fig. S3), which is consistent with the report that *L*. *fermentum* I5007 could slightly decrease feed conversion in piglets^32^. The decreased feed conversion in HCM2 and HED1 mice may due to the increased ratio of *Firmicutes* to *Bacteroidetes* (Fig. S4), which has been reported to be associated with improved energy-harvesting capacity^33^. Furthermore, *L*. *reuteri* HCM2 significantly increased the villus/crypt ratio in mice, which indicates that the absorption of nutrients might be increased by feeding mice *L*. *reuteri* HCM2.

The healthy colon is usually dominated by obligate anaerobes, while dysbiosis is often associated with a sustained increase in the abundance of facultative anaerobic *Proteobacteria*, which results in the disruption of anaerobiosis^34^. For example, Zhang *et al*. found that ETEC infection could increase the relative abundance of *Proteobacteria* in newly weaned pigs compared with the uninfected control^35^. In our study, we analyzed the dynamics of gut microbiota and found that the abundance of *Proteobacteria* gradually increased after ETEC infection (ED1 and ED3). By contrast, not much difference in *Proteobacteria* abundance was observed in the *L*. *reuteri* HCM2 pre-supplementation groups (HED1 and HED3). Higher levels of *Enterobacteriaceae* (belonging to *Proteobacteria*) were detected in mice without *L*. *reuteri* HCM2 pre-supplementation, and this may contribute to a thinner and more penetrable mucus layer, which increases the risk of chronic colitis^36^. ETEC are members of *Enterobacteriaceae*, and there was a significant difference in the relative abundance of *Enterobacteriaceae* between the Control and HCM2 groups. It is interesting that there was no big difference in the relative abundance of *Enterobacteriaceae* between the ED1 and HED1 and groups (p > 0.05). This might be due to the amount of ETEC challenge. However, after 3 days of recovery, the relative abundance of *Enterobacteriaceae* in ED3 was significantly higher than that in the group HED3 (p = 0.0397). It is clear that HCM2 pre-supplementation decreases the relative abundance of *Enterobacteriaceae* and may prevent the dysbiosis of gut microbiota caused by ETEC.

It is noteworthy that the relative abundance of *Helicobacter* was increased by ETEC infection, while there was no obvious difference in the abundance of *Helicobacter* in the *L*. *reuteri* HCM2 pre-supplementation groups (HED1 and HED3) after ETEC infection. It has been reported that many cases of non-*H*.*pylori Helicobacter* (NHPH) infection in humans occur in immunocompromised patients, because NHPH can induce high levels of inducible nitric oxide synthase and the development of DNA double-stranded breaks, but the properties of *Lactobacillus* can prevent *Helicobacter* infection or its related pathologies^37^. In our study, *L*. *reuteri* HCM2 supplementation not only maintained the abundance of *Helicobacter* at a relatively low level, but also increased the relative abundance of *Lactobacilli*. It has been reported that the abundance of *Helicobacteraceae* in a rat model of colon cancer was decreased by administering a probiotic cocktail (*Lactobacillus acidophilus*, *Bifidobacteria bifidum*, and *Bifidobacteria infantum*), which promoted the growth of *Lactobacilli* and thus altered the gut microbiota^38^. We conclude that pre-supplementation with *L*. *reuteri* HCM2 may alter the gut microbiota and protect against ETEC and the increase in abundance of detrimental bacteria caused by ETEC.

We monitored the dynamic changes in dominant genera in each group of mice and intriguingly found that ETEC could reduce the relative abundance of *Lactobacillus* in ED1 and HED1 mice. The relative abundance of *Lactobacillus* in the ED3 group was lower than that of both the Control and ED1 groups, while in the HED3 group, the abundance of *Lactobacillus* was the same as that in the control, suggesting that *L*. *reuteri* HCM2 pre-supplementation prevented the reduction in *Lactobacillus* abundance. It was reported that *lactobacilli* abundance in the colon could be reduced by ETEC, while pre-administration of probiotics could increase the abundance of *lactobacilli* and enhance goblet cell function to ameliorate enteritis^35^. *Lactobacilli*, which are considered health promoting probiotics^39^, might promote defense against detrimental bacteria in the gut by creating an acidic environment (e.g., pH 4.5), synthesizing exopolysaccharides^5^, competitively excluding intestinal pathogens^40^, improving antioxidant activity^19^, or activating and enhancing local cell-mediated immunity against certain enteric pathogens^41^. So, stabilizing the relative abundance of *Lactobacillus* is important for maintaining the balance of gut microbes. The relative abundances of the dominant genera *Alistipes*, *Bacteroides* and *Alloprevotella*, which belong to the *Bacteroidetes*, were also higher after ETEC infection, but the abundances returned back to the pre-infections after 3 days of recovery (HED3). This also indicates that *L*. *reuteri* HCM2 pre-supplementation could stabilize the relative abundance of *Bacteroidetes*. In conclusion, *L*. *reuteri* HCM2 pre-supplementation may modulate the gut microbiota, preventing the dysbiosis of intestinal microbiota caused by ETEC.

## Materials and Methods

### Bacterial strains and cell lines

*E*. *coli* F4-producing strain W25K (ETEC; O149:K91, K88ac; LT, STb, EAST)^4,42^ was used and cultured in LB medium. *Lactobacillus reuteri* HCM2 was isolated from a 6-week-old healthy piglet and was anaerobically cultured in MRS medium. All strains were sub-cultured twice prior to being used for experiments. The Caco-2 cell line was cultivated in complete Dulbecco’s modified Eagle’s minimal essential medium (DMEM, C12430500BT, purchased from Thermo Fisher Scientific (China) Co., Ltd.) supplemented with heat-inactivated fetal bovine serum (10% v/v) and 100 U/ml penicillin-Streptomycin. The medium was replaced every 2 days. Cells were grown at 37 °C, 5% CO_2_/95% air in T25 flasks.

### Animals, feeding procedures, and infection

**Figure 6:**
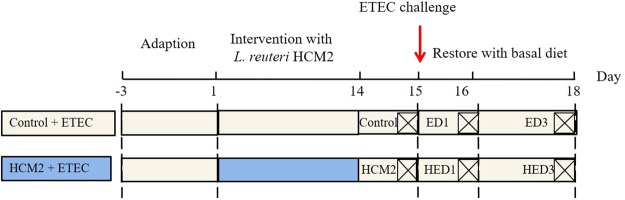
A schematic of the experimental design. The gray boxes indicate that mice received a common diet, and blue boxes indicate that mice were treated with *L*. *reuteri* HCM2. The red arrow indicates when mice were challenged with ETEC. Control: mice received a basal diet. HCM2: mice received a basal diet and 10^9^ CFUs *L*. *reuteri* HCM2 daily for two consecutive weeks. ED1: mice received a basal diet, were challenged with 10^8^ CFUs ETEC by intragastric administration at day 15, then received a basal diet for 1 day. HED1: mice received a basal diet and 10^9^ CFUs *L*. *reuteri* HCM2 daily for two consecutive weeks, were challenged with 10^8^ CFUs ETEC by intragastric administration at day 15 and then received a basal diet for 1 day. ED3: mice received a basal diet, were challenged with 10^8^ CFUs ETEC by intragastric administration at day 15, then received a basal diet for 3 days. HED3: mice received a basal diet and 10^9^ CFUs *L*. *reuteri* HCM2 daily for two consecutive weeks, were challenged with 10^8^ CFUs ETEC by intragastric administration at day 15, and then received a basal diet for 3 days.

ICR^6,7,42^ male mice (5 weeks of age) were purchased from SLACCAS (Shanghai Laboratory Animal Center). Only male mice were used to avoid differences in microbiota composition resulting from sex and maternal factors^43^. Animal experiments were approved by the Laboratory Animal Ethical Commission of the Chinese Academy of Sciences and performed according to its guidelines. In order to test the protective effect of *L*. *reuteri* HCM2 against ETEC *in vivo*, we developed a mouse model as described in a previous study^6,35^ with minor modifications. The mice were housed in a pathogen-free mouse colony (temperature, 25 ± 2 °C; relative humidity, 45–60%; lighting cycle, 12 h/d). After acclimatization for three days, the mice were randomly divided into six groups (n = 5 for each group) as shown in Fig. 6. The Control group received a basal diet^44^; the HCM2 group received a basal diet and a 200 μL suspension of 5 × 10^9^ CFU/ml *L*.*reuteri* HCM2 daily^45^ for two consecutive weeks; the ED1 (basal diet + ETEC + Day 1) group and the ED3 (basal diet + ETEC + Day 3) group received a basal diet, were challenged with 100 μL of 10^9^ CFU/ml ETEC^6,7^ by intragastric administration at day 15, then received a basal diet for 1 and 3 days, respectively; the HED1 (HCM2 + ETEC + Day 1) group and the HED3 (HCM2+ ETEC+ Day 3) group received a basal diet and 10^9^ CFUs *L*. *reuteri* HCM2 daily for two consecutive weeks, were challenged with 10^8^ CFUs ETEC by intragastric administration at day 15, then received a basal diet for 1 and 3 days, respectively. Mice were intragastrically administered *L*. *reuteri* HCM2 or ETEC via 12-gauge gavage needles (Bio-Medical Needles, Beijing Solarbio Science & Technology Co., Ltd.). Control and HCM2 mice were sacrificed at D15, ED1 and HED1 mice were sacrificed at D16, and ED3 and HED3 mice were sacrificed at D18. All mice were sacrificed at 10:00 am and samples were collected. Briefly, after cervical dislocation, blood was collected by removing the eyeball, then the jejunum, the contents of the jejunum and the contents of colon were collected. The body weights and food intake of the mice were regularly monitored during the experiment.

### Antimicrobial activity assay

ETEC were cultured overnight in Shake-tubes and were standardized to OD_600_ = 2. The supernatants of the *L*. *reuteri* HCM2 overnight cultures were filtered through a 0.22 μm filter, and 100 μl of CFS was placed into each well of an ETEC indicator strain agar plate. MRS medium was tested as a negative control. After incubation at 37 °C for 24 h, the diameters of the inhibition zones were measured. To further validate the capacity of *L*. *reuteri* HCM2 to antagonize ETEC, ETEC cells at a density of 1 × 10^6^ CFUs/ml were seeded into the wells of a 96-well microtiter plate, and 0%, 5%, 10% or 20% (v/v) CSF of *L*. *reuteri* HCM2 was added to each well. The absorbance of ETEC at 600 nm was monitored every 2 h.

### Scanning electron microscopy

SEM experiments were performed as described by Aiba *et al*. with slight modifications^46^. Briefly, a 10^8^ CFUs/ml inoculum of ETEC was treated with 20% *L*. *reuteri* HCM2 CFS for 12 h at 37 °C, and a non-treated ETEC culture served as a control. After washing with fresh PBS, cells were fixed with 2.5% glutaraldehyde for 12 h. The bacteria were then washed with demineralized water 3 times, 6 min each time. After dehydration with ethanol (stepwise gradient of 50%, 70%, 85% 95% and 100%), the specimens were freeze-dried in a critical-point dryer (Czech FEI company) and coated with gold. The morphology of the cells was visualized by examining the specimens with a Hitachi cold field emission scanning electron microscope.

### Characterization of the tolerance of *L*. *reuteri* HCM2

*L*. *reuteri* HCM2 cells from an overnight culture were washed twice with PBS (pH 7.2), and the concentration was adjusted to about 10^8^ to 10^9^ CFUs/ml. One hundred microliters of cells were transferred to 900 μl of artificial gastric juice (pH 2.5)^47^ or artificial small intestine fluid (pH 6.8) and incubated anaerobically for 2 h at 37 °C. After recovery for 48 h, viable bacterial cells were counted by plating serial dilutions of the culture in PBS on MRS medium.

### *In vitro* adhesion assay

Caco-2 cells were prepared on a Millicell EZ SLIDE 4-well and seeded at a concentration of 10^5^ cells per well. Caco-2 cells were cultured for 15 days in cell culture medium to obtain confluence, and the medium was changed on alternate days. Caco-2 cells were washed with D-Hanks buffer solution and fresh culture medium without antibiotic solution was added 48 h before the adhesion assay^40^. The overnight *L*. *reuteri* HCM2 culture was washed with D-Hanks buffer solution and then re-suspended in fresh DMEM. The *L*. *reuteri* HCM2 suspension was diluted to 10^8^ CFUs/ml and 1 ml was added into the Millicell EZ SLIDE 4-well. After co-culturing for 2 h, the Caco-2 monolayers were washed three times and fixed for 20 min at room temperature with 4% paraformaldehyde (w/v) fix solution. The Caco-2 monolayers were then washed five times with D-Hanks buffer solution, air-dried and Gram stained^48^. The images of *L*. *reuteri* HCM2 binding to Caco-2 cells were captured under microscope.

The amount of *L*. *reuteri* HCM2 binding to Caco-2 cells was further quantified as described by Todoriki *et al*. with slight modifications^49^. Briefly, the Caco-2 cells were cultivated in 6-well plates until confluence was reached and then used for adhesion experiments. Caco-2 monolayers were washed twice with PBS, and 3 ml bacterial suspension (5 × 10^8^ cells/ml) was added to each well of the tissue culture plate. The plates were incubated at 37 °C in 5% CO_2_/95% air. After 2 h of incubation, the monolayers were washed three times with PBS. Following the last wash, Caco-2 cell monolayers were covered with 3 ml distilled water and mechanically agitated by vigorous pipetting to suspend the Caco-2 cells and bacteria. Adherent bacteria were serial diluted by 10-fold and plated on MRS media. The number of *L*. *reuteri* HCM2 colonies were counted after anaerobic incubation for 48 h.

### Competition and displacement assays

Caco-2 cells were cultivated in 6-well plates to 80% confluence and then used for adhesion experiments^40,50^. Suspensions of *L*. *reuteri* HCM2 and ETEC were prepared with DMEM medium. For the competition assays, bacterial cells were washed twice with PBS, and of 100 μl of *L*. *reuteri* HCM2 suspension (10^8^ CFUs) and 100 μl of ETEC suspension (10^8^ CFUs) were added to each well simultaneously. The cultures were incubated at 37 °C for 90 min, then the monolayers were washed twice with PBS and digested with 100 μl trypsin (0.25%) for 2 min. For displacement assays, 100 μl ETEC suspension (10^8^ CFUs) was added to each well, and the samples were incubated for 45 min. Then 100 μl of *L*. *reuteri* HCM2 suspension (10^8^ CFUs) was added, and the samples were incubated for another 45 min. The plates were incubated and digested as described above. For both assays, serial dilutions of the adherent bacteria were plated on MacConkey Agar containing 50 μg/ml streptomycin and incubated at 37 °C for 16 h. The number of colonies were then counted. The ETEC strain used in this study is resistant to streptomycin.

### Morphological analyses

Mouse jejunums were fixed with 4% paraformaldehyde-PBS overnight and then dehydrated and embedded in paraffin blocks. Sections (5 μm) from these blocks were deparaffinized, hydrated, and then stained with hematoxylin and eosin (H&E). At least three villus lengths and crypt depths per slide were measured using Image-Pro Plus software 6. Five mice were studied from each group. The data collectors were unaware of the treatment status of the examined slides.

### Detection of serum IgG and IgA

Blood (500 μl) was collected from each mouse. Serum was collected after centrifugation at 4500 rpm for 10 min at 4 °C and stored at −20 °C until IgG and IgA were quantified. Total IgG and IgA in the serum were measured by enzyme-linked immunosorbent assay (Mouse IgG ELISA Kit and Mouse IgG ELISA Kit, AMEKO). The concentrations were then calculated from standard curves.

### Illumina HiSeq sequencing and data processing

Total genomic DNA was extracted from the colonic contents of mice from the six groups using the Qiagen QIAamp DNA Stool Mini Kit. The V3-V4 hypervariable region of the bacteria 16 S rRNA gene was amplified using the primers 338 F 5′-ACTCCTACGGGAGGCAGCA-3′ and 806 R 5′-GGACTACHVGGGTWTCTAAT-3′. PCR products were mixed in equidensity ratios and purified with the Qiagen Gel Extraction Kit (Qiagen, Germany). Sequencing libraries were generated using the TruSeq® DNA PCR-Free Sample Preparation Kit (Illumina, USA), and index codes were added. The library quality was assessed using the Qubit@ 2.0 Fluorometer (Thermo Scientific) and the Agilent Bioanalyzer 2100 system. Finally, the library was sequenced on the Illumina HiSeq2500 platform, and 250 bp paired-end reads were generated, assigned to samples based on their unique barcode and truncated by cutting off the barcode and primer sequences. Paired-end reads were merged using FLASH^51^, which was designed to merge paired-end reads when at least some of the reads overlap with the read generated from the opposite end of the same DNA fragment, and the trimmed sequences were called raw tags. The raw tags were filtered using the QIIME quality control process to obtain high-quality clean tags^51^. The tags were compared with the reference database (Gold database) using the UCHIME algorithm to detect chimeric sequences, which were later removed^52,53^. The effective tags were finally obtained.

### Bioinformatics analysis

Sequence analysis was performed using Uparse software (Uparse v7.0.1001). Sequences sharing greater than 97% similarity were assigned to the same OTUs^54^. A representative sequence for each OTU was selected for further annotation. For each representative sequence, the GreenGene Database was used to obtain taxonomic information using the RDP classifier algorithm^55,56^. OTU abundance was normalized to the number of sequences in the sample with the fewest sequences. Subsequent analysis of alpha diversity and beta diversity were performed using the normalized data. Species diversity complexity of a sample was analyzed using six indices of alpha diversity, including Observed-species, Chao1, Shannon, Simpson, ACE and Good-coverage. All indices were calculated with QIIME (Version 1.7.0) and visualized using R software (Version 2.15.3). The differences in dominant bacterial communities between groups were determined based on LDA Effect Size.

### Statistical analysis

Data shown are the means ± SD or SEM. Differences in the means between two groups were analyzed by performing an unpaired t test (Prism 7.0) if the data were normally distributed and the samples had equal variance, or by performing a non-parametric test (Mann–Whitney U test, Prism 7.0) if the data were not normally distributed. Means of more than two groups were analyzed by performing one-way ANOVA followed by the Dunnett multiple comparisons test (Prism 7.0) if the data were followed a Gaussian distribution and had equal variance, or by performing the Kruskal-Wallis test followed by Dunn’s multiple comparisons test (Prism 7.0) if the data were not normally distributed. The Kolmogorov-Smirnov test (Prism 7.0) was used to determine if the data followed a Gaussian distribution. The homogeneity of variance test (SPSS 22.0) or the Brown-Forsythe test (Prism 7.0) was used to test for equal variance. Differences with p < 0.05 were considered significant.

## Electronic supplementary material

Supplementary Information
