# Supplementary material for: Lactobacillus reuteri HCM2 protects mice against Enterotoxigenic Escherichia coli through modulation of gut microbiota
Source: Sci Rep. 2018 Nov 30;8:17485. doi: 10.1038/s41598-018-35702-y (PMC6269427; doi:10.1038/s41598-018-35702-y)
Supplement: Supplementary file 1 — Supplementary Information [file 41598_2018_35702_MOESM1_ESM.docx]

***Lactobacillus reuteri* HCM2** **protects mice against Enterotoxigenic *Escherichia coli* through** **modulation of** **gut microbiota**

Tianwei Wang^1,2^†, Kunling Teng^1^†, Gang Liu^3^, Yayong Liu^1,2^, Jie Zhang^1,2^, Xin Zhang^4^, Min Zhang^4^, Yong Tao^1,2^, Jin Zhong^1,2*^

^1^ State Key Laboratory of Microbial Resources, Institute of Microbiology, Chinese Academy of Sciences, Beijing 100101, China

^2^ University of Chinese Academy of Sciences, Beijing 100101, China

^3^ Key Laboratory of Agro-Ecological Processes in Subtropical Region, Institute of Subtropical Agriculture, Chinese Academy of Sciences, Hunan 410125, China

^4^ LongDa Foodstuff Group Co., Ltd, Shandong Province, 265231, China

**Figure S1.** The rarefaction curves and rank abundance curves.


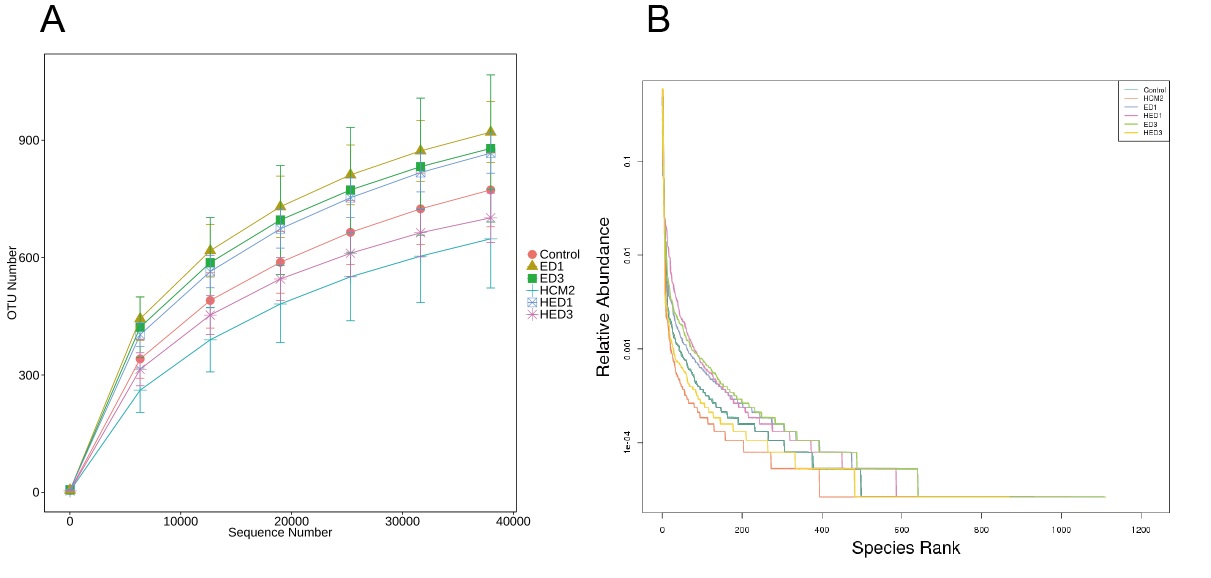


**Figure S2**. Bacteria composition differs from the ED3 and HED3. A: Taxonomic cladogram obtained from LEfSe sequence analysis (n=5). Biomarker taxa are highlighted by colored circles and shaded areas. Each circle’s diameter reflects the abundance of the taxa in the community. B: The taxa whose abundance differed between the HED3 mice and the ED3 challenged mice are indicated (n=5). The cutoff value of ≥4 used for the linear discriminant analysis (LDA) is shown.


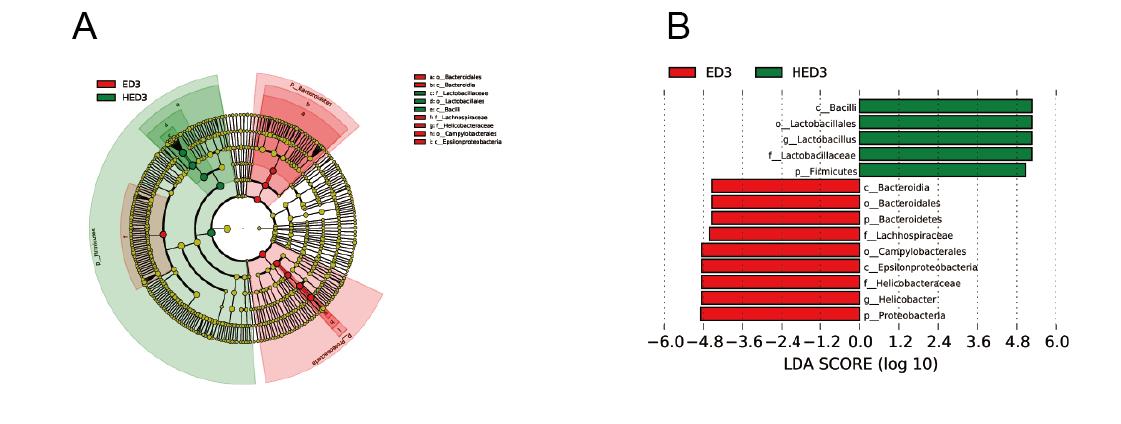


**Figure S3.** The feed conversion of mice. Feed conversion was estimated by feed intake (g) divided by weight gain (g)


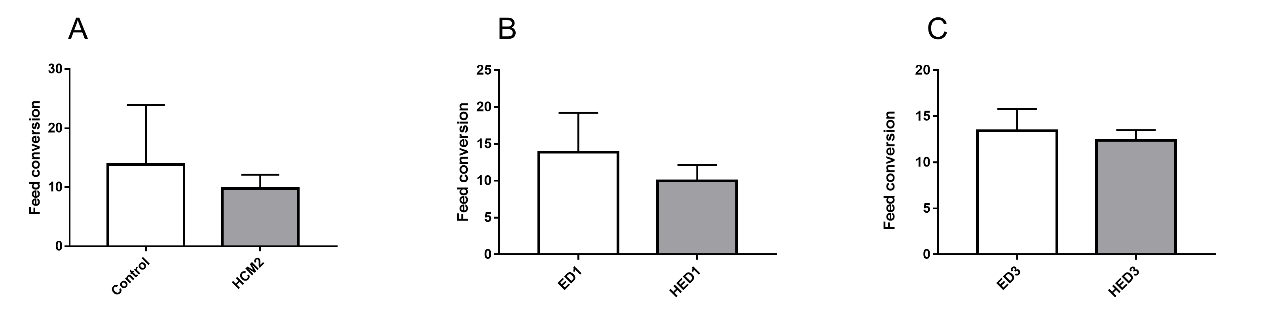


**Fig****ure S4.** The ratio of *Firmicutes* to *Bacteroidetes.* *, significant difference at p < 0.05.


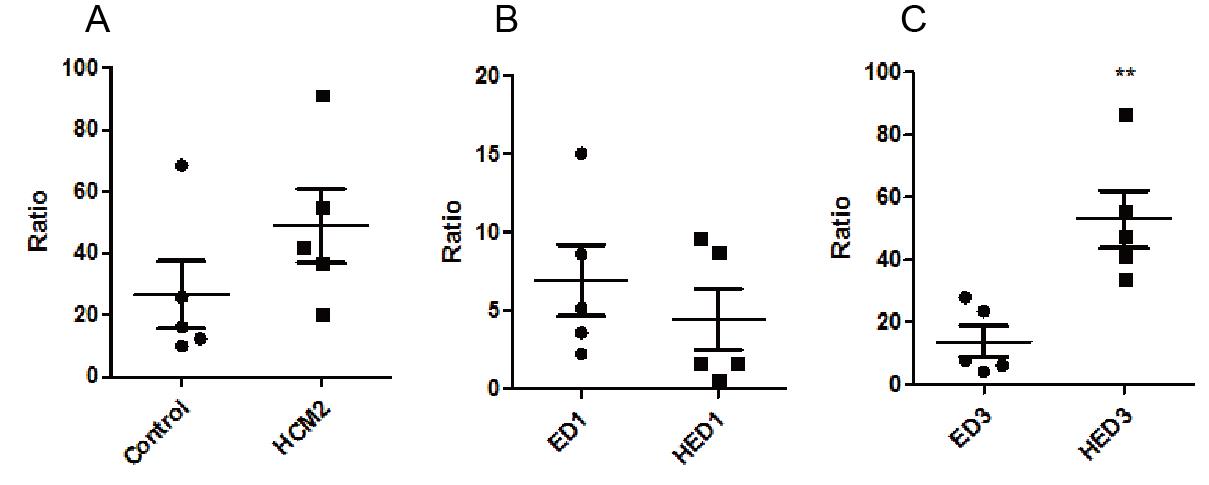


Table S1 Pyrosequencing data and diversity indices of the colonic microbiota of mice.

| **Group** | **Coverage** | **Richness estimator** | | |  | **Diversity index** | |  |
| --- | --- | --- | --- | --- | --- | --- | --- | --- |
|  |  | **Chao1** | **OS** | **ACE** |  | **Shannon** | **Simpson** | **PD** |
| **D15** | | | | | | | | |
| Control | >99% | 1003.4±139.8 | 773±105.4 ^ab^ | 1038.7±126.5 |  | 3.4±0.8 ^ab^ | 0.7±0.15 ^ab^ | 65±8.7 ^ab^ |
| HCM2 | >99% | 858.3±141.5 | 648±140.6 ^b^ | 896.7±160.7 |  | 2.6±0.5 ^b^ | 0.61±0.12 ^b^ | 56.1±9.2 ^b^ |
| **D16** | | | | | | | | |
| ED1 | >99% | 1096.7±86.2 | 920.6±87.2 ^a^ | 1146.9±85 |  | 4.4±0.4 ^a^ | 0.82±0.04 ^a^ | 73.4±7.8 ^a^ |
| HED1 | >99% | 1110.9±87.2 | 867±57.4 ^ab^ | 1140.9±63.9 |  | 4.9±0.3 ^a^ | 0.87±0.07 ^a^ | 72.4±1.8 ^ab^ |
| **D18** | | | | | | | | |
| ED3 | >99% | 1076.3±286.4 | 879±94.1 ^ab^ | 1104.4±298 |  | 4.4±1.2 ^a^ | 0.82±0.12 ^a^ | 68.4±14.5 ^ab^ |
| HED3 | >99% | 853.2±104.5 | 701.6±70.7 ^ab^ | 875.5±88.9 |  | 2.8±0.3 ^b^ | 0.61±0.06 ^b^ | 58.3±4.2 ^ab^ |

The coverage percentage, richness estimators (Chao1, OS and ACE) and diversity indices (Shannon and Simpson) were calculated using the mothur program. PD: phylogenetic distance (whole tree); OS: observed species. Data in the same row that do not share a common superscript are significantly different (P < 0.05).
